# A Comprehensive Review of Glucose Biosensors Based on Nanostructured Metal-Oxides

**DOI:** 10.3390/s100504855

**Published:** 2010-05-12

**Authors:** Md. Mahbubur Rahman, A. J. Saleh Ahammad, Joon-Hyung Jin, Sang Jung Ahn, Jae-Joon Lee

**Affiliations:** 1 Department of Advanced Technology Fusion, Konkuk University, Seoul 143-701, Korea; E-Mails: mahbub@konkuk.ac.kr (M.M.R.); saleh203@konkuk.ac.kr (A.J.S.A.); 2 KFnSC Center, Konkuk University, Seoul 143-701, Korea; E-Mail: jhjin@konkuk.ac.kr; 3 Korea Research Institute of Standard and Science, Yuseong, Daejeon 305-340, Korea; E-Mail: sjahn@kriss.re.kr; 4 Department of Applied Chemistry, Konkuk University, Chungju 380-701, Korea

**Keywords:** nanostructured metal-oxides, glucose biosensor, electrochemical principles, enzymatic sensor, nonenzymatic sensor

## Abstract

Nanotechnology has opened new and exhilarating opportunities for exploring glucose biosensing applications of the newly prepared nanostructured materials. Nanostructured metal-oxides have been extensively explored to develop biosensors with high sensitivity, fast response times, and stability for the determination of glucose by electrochemical oxidation. This article concentrates mainly on the development of different nanostructured metal-oxide [such as ZnO, Cu(I)/(II) oxides, MnO_2_, TiO_2_, CeO_2_, SiO_2_, ZrO_2,_ and other metal-oxides] based glucose biosensors. Additionally, we devote our attention to the operating principles (*i.e.*, potentiometric, amperometric, impedimetric and conductometric) of these nanostructured metal-oxide based glucose sensors. Finally, this review concludes with a personal prospective and some challenges of these nanoscaled sensors.

## 1. Introduction

Diabetes mellitus is one of the principal causes of death and disability in the World, and is highly responsible for heart disease, kidney failure, and blindness. About 200 million people in the world are afflicted with diabetes mellitus. This figure is expected to rise up to more than three hundred million by 2030 [1]. Frequent testing of physiological blood glucose levels to avoid diabetic emergencies, is crucial for the confirmation of effective treatment [2–5]. Therefore, the development of high sensitive, low-cost, reliable glucose sensors having an excellent selectivity has been the subject of concern for decades, not only in medical science but also in the food industries [6,7]. Glucose oxidase (GOx)-based glucose biosensors have prevalently had a hold on the glucose sensor research and development over the last four decades and the market place as well. This is due to the high demand of sensitive and reliable blood glucose monitoring in biological and clinical aspects [8–11]. There are still some disadvantages of enzyme-based glucose determination. Examples include complicated enzyme immobilization, critical operating conditions such as optimum temperature and pH, chemical instability, and high cost [12,13].

The historical commencement of biosensors was in 1960s with the pioneering work of Clark and Lyons [14], and the first enzyme-based glucose sensor commenced by Updike and Hicks in 1967 [15]. Since then, an extensive research have been done on the amperometric, potentiometric, and impedimetric or conductometric glucose biosensors based on the GOx [16–23], that catalyzes the oxidation of glucose to produce gluconic acid as shown in equation (1):

(1) $$D-\text{glucose}+O_{2}+H_{2}O\overset{\text{GOx}}{\to }D-\text{gluconic acid}+H_{2}O_{2}$$

The activity of enzymes is obviously affected by the temperature, pH, humidity, and toxic chemicals [24]. To solve these problems, many enzyme-free sensors have been investigated to improve the electrocatalytic activity and selectivity toward the oxidation of glucose. This can be done by using: (1) inert metals such as Pt [25–27], Au [28–30] and Ni [31,32]; (2) metal alloys containing Pt, Au, Pb, Ir, Ru, Cu, and Pd [2,33–37]; and (3) metal-dispersed carbon nanotubes (CNTs) framework in which Pt, Pb, Pd, or Au are mixed with CNTs to form nanocomposites [38–42]. However, these materials are unsatisfactory with regards to sensitivity and selectivity, are high costing, and show quick loss of activity by adsorption and accumulation of intermediates or chloride ions [2,43]. Therefore, the development of highly selective, sensitive, inexpensive, reliable and fast enzymatic/nonenzymatic glucose sensor is still imperatively needed.

In recent years, an increasing number of researchers have explored the production of novel nano-scale metal oxides, noble metal-doped metal oxides, metal oxide-CNTs nanocomposites, and metal oxide-polymer composites. Novel analytical devices based on nanostructured metal oxides are cost-effective, highly sensitive due to the large surface-to-volume ratio of the nanostructure, and additionally show excellent selectivity when coupled to biorecognition molecules with simple design [44–47]. Some metal oxides such as ZnO and CeO_2_ show high isoelectric point (IEP), excellent biocompatibility, and easy synthetic procedure for nanostructure that enables reliable immobilization of GOx. On the other hand, MnO_2_ and ZrO_2_ having low IEP values are suitable for the immobilization of high IEP proteins. The catalytic ability of transition metal oxides such as CuO and NiO of nonenzymatic direct electrooxidation of glucose is one of the attractive properties in glucose detection allowing minimum fabrication cost and stable glucose sensors. This article provides a comprehensive review of the state-of-the-art research activities that focus on several important metal-oxide nanostructures and nanocomposites in addition to the application of nanostructured metal oxides to glucose sensing. Also, the most commonly-used electrochemical detection methods for the glucose sensing will be discussed.

## 2. Electrochemical Principles of Glucose Biosensors

There is no doubt that the development of an ideal glucose sensor must be top issue for the biosensor industry. Numerous processes and methodologies have been developed for creating new glucose biosensors such as electrochemical methods [48], colorimetry [49], conductometry [50], optical methods [51], and fluorescent spectroscopy [52]. Among them, the electrochemical glucose sensors have attracted the most attention over the last 40 years because of their unbeaten sensitivity and selectivity. Additionally, electrochemical techniques show lower detection limit, faster response time, better long term stability and inexpensiveness. The extremely sensitive fluorescent spectroscopy can even detect a single molecule and a few fluorescence-based *in vivo* monitoring of glucose are available, but none of them are practically applicable to diabetes management [53]. The electrochemical glucose sensors are basically categorized into three major groups depending on the measurement principles: *i.e.*, potentiometric, amperometric, impedemetric or conductometric sensors.

### 2.1. Potentiometric Glucose Sensor

Potentiometry is commonly used to measure glucose concentrations greater than 10^−5^ M, which is in the physiological range in most cases. The potential difference between the reference electrode and the indicator electrode is measured at zero current flow. The ideally nonpolarizable reference electrode provides a constant potential, while the indicator electrode shows an erratic potential depending on the concentration of the analytes. The zero current potentials applied between those two electrodes are recorded as a function of the concentrations of target analytes in a logarithmic manner [48]. The potential difference at the electrode-electrolyte interface arising from unbalanced activities of species *i* in the electrolyte phase (*s*) and in the electrode phase (*β*) is related by the following Nernst equation:

(2) $$E=E_{o}+\frac{\mathrm{RT}}{Z_{i}F}\;\text{ln}\;\frac{a_{i}^{s}}{a_{i}^{\beta }}$$

where *E_0_* is the standard electrode potential, *R* the gas constant, *T* the absolute temperature, *F* the faraday constant, *a_i_* the activity of species *i*, and *Z_i_* the number of moles of electron involved.

**Figure 1.**
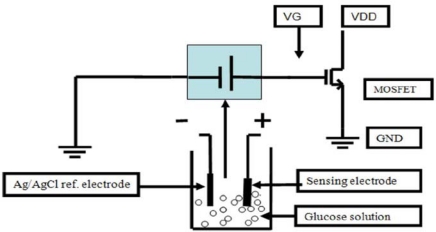
Schematic illustration of the configuration of the MOSFET-based potentiometric glucose detection using an extended-gate functionalized-ZnO nanowire as a working electrode and the Ag/AgCl reference electrode (reproduced with permission from [54]. Copyright 2009, IEEE).

Potentiometric sensors are divided into the metal-oxide sensitive field effect transistor (MOSFET), the light-addressable potentiometric sensor (LAPS), the ion-sensitive field effect transistors (ISFET) and the ion-selective electrodes (ISE). Ali *et al.* developed a commercial MOSFET using a GOx modified ZnO nanowire. GOx-functionalized ZnO nanowires were grown on the Ag wire and then directly connected to the MOSFET gate (Figure 1) [54]. They tested the response time and the stability of the MOSFET sensor by using three different GOx/ZnO modified Ag electrodes, *i.e.*, vertically aligned, uniform nonaligned, and nonuniform nonaligned ZnO nanowires. The results showed that well aligned ZnO-modified electrode displayed a good stability, short response time (<100 ms), and improved detection limit. They also further demonstrated that the GOx/ZnO modified MOSFET is able to be used for the immobilization of other biomolecules to make versatile electrodes for biosensing.

ISFETs and LAPS have attracted much attention for biosensing application being especially convenient for construction. The principles of LAPS are based on the activation of semiconductor by a light emitting diode [55]. Seki *et al.* developed a LAPS based on SiO_2_/Al_2_O_3_ film grown on an n-type Si substrate. The GOx was immobilized on the film at various pH in the range of 3 to 11. Upon exposure to the light emitting diode, the equilibrium potential of the GOx-modified SiO_2_/Al_2_O_3_ film was increased linearly with the increase of glucose concentration up to 4 mM. An increased sensitivity was also observed by introducing hexacyanoferrate (III) as an alternative to oxygen because the reduced form of flavin adenine dinucleotide (FADH_2_) positioned within the active site of the GOx is more easily oxidized with hexacyanoferrate (III) [56]. While, the principle of ISFET is based on the local potential generated by surface ions from a solution [55]. Luo *et al.* built up a glucose sensitive enzymatic field effect transistor (ENFET) by modifying the gate surface of the ISFET with SiO_2_ nanoparticles and GOx. The SnO_2_ based glucose sensor showed a good stability and reproducibility with a detection limit of *ca.* 0.025 mM [57]. A few reports are available on nonenzymetic potentiometric glucose sensor based on poly(aniline boronic acid) suggested by Shoji and Freund [58,59]. These sensors showed improved sensitivity for fructose compared to glucose.

### 2.2. Amperometric Glucose Sensor

Amperometry is a quite sensitive electrochemical technique in which the signal of interest is current that is linearly dependent on the target concentration by applying a constant bias potential. Glucose is oxidized at the working electrode composed of a noble metal formed by either the physical vapor deposition or screen-printing and the biorecognition species such as GOx for glucose sensing [60]. An amperometric biosensor comprises two or three electrodes. The former consists of a reference and a working electrode. Application of the two-electrode system to biosensors is limited, because at high current flow the potential control becomes difficult as a result of sizable IR drop. Instead, the third electrode is commonly introduced as an auxiliary counter electrode having a large surface area to make most of current flows between the counter and the working electrodes, though voltage is still applied between the working and the reference electrodes.

**Figure 2.**
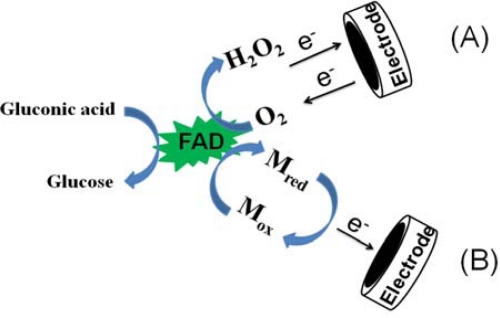
**(A)** A schematic illustration of the first generation and **(B)** the second generation amperometric glucose sensors (redrawn from reference [61]).

There are three modes of the glucose oxidation referred to as the first, the second and the third generation glucose sensors depending on the electron transfer mechanisms. The nanostructured metal-oxide based glucose sensor belong to the third generation. Figure 2(A) depicts the first generation glucose sensor where oxygen is used as a mediator between the electrode and the GOx. The oxygen is reduced to form hydrogen peroxide in the presence of glucose by flavin adenine dinucleotide (FAD), a prosthetic group of GOx, and FADH_2_ redox couple. The reduction rate of the oxygen is proportional to the glucose concentration that is quantified by either measuring the augmentation of hydrogen peroxide or decrement of the oxygen concentration [5,61]. On the other hand, artificial electron mediators (M), e.g., ferro/ferricyanide, hydroquinone, ferrocene, and various redox organic dyes between the electrode and the GOx are employed in the second generation glucose sensor. These mediators make the electron transfer rate between the electrode and the GOx faster and also give a way of getting around for a case when limited oxygen pressure commonly observed from the first generation glucose sensor [62,63]. Figure 2(B) represents the second generation glucose sensor, where M_ox_ and M_red_ are the oxidized and reduced forms of mediator, respectively. The reduced form of flavin group (FADH_2_) of GOx is reoxidized in presence of glucose by the reduction of M_ox_ to form M_red_. The output current signal generated, when further oxidation of M_red_ occurs at the electrode surface, is proportional to the glucose concentration [5,62,63].

**Figure 3.**
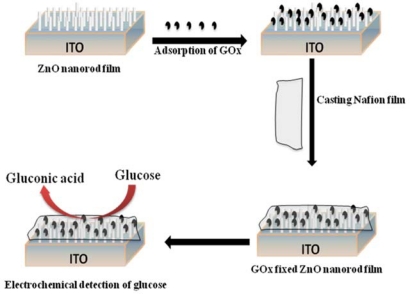
Schematic illustration of the preparation of the third generation amperometric glucose sensor based on the GOx-immobilized aligned ZnO nanorod (redrawn from reference [69]).

In the third generation glucose sensor, the GOx is directly coupled to the electrode. The direct electron transfer efficiently generates an amperometric output signal. The improved sensing performance by the direct electron transfer has been realized by incorporating the enzyme with metal nanoparticles [64,65], and semiconductive nanomaterials [66,67]. The nanocrystalline metal-oxide plays a vital role in the enzyme immobilization owing to its highly specific surface area, good biocompatibility and stability [68]. Liu *et al.* fabricated a novel third generation amperometric glucose sensor based on the aligned ZnO nanorod formed on an ITO electrode (Figure 3). The immobilized GOx on the ZnO nanorod shows still high catalytic activity, effective protection by Nafion (perfluorosulfonate ionomer) membrane cast on the film, a wide linear range with good selectivity and freedom from the interference effects of uric acid and ascorbic acid in real samples [69].

In recent years progressive attempts have been made to determine the glucose without any enzyme for the reliable fast determination [70–72]. Most enzymeless electrochemical glucose sensors rely on the properties of the electrode materials, on which the glucose is oxidized directly. Carbon, platinum, gold and nanostructured CuO-modified CNTs have been widely investigated as candidates for improving the sensing performance of enzymeless sensors [73,74]. However, some problems including poor selectivity and low sensitivity due to the surface poisoning by the adsorbed intermediates or chloride ions still remain [75].

### 2.3. Impedimetric/Conductometric Glucose Sensor

The impedimetric biosensor is less frequently used as compared to the potentiometric and the amperometric ones. Electrochemical impedance spectroscopy (EIS) is a powerful analytical tool which allows us to effectively visualize the actual electrical double layer structure of a modified electrode [76] although recording of an impedance spectrum within a broad frequency range is time consuming. The glucose does not affect the dielectric spectrum in the MHz frequency region [77,78] and direct detection of the glucose is available. The expression of impedance in a simple RC circuit is as follows:

(3) $$Z^{2}=R^{2}+\frac{1}{{\left(2fC\right)}^{2}}$$

where *Z* is impedance, R the resistance and C the capacitance.

Conductance is the reciprocal of resistance, so sometimes the impedimetric biosensor is also called a conductometric biosensor [78]. Shervedani *et al.* developed a quantitative method for the determination of glucose based on the EIS measurements. In this method, the GOx was immobilized on the mercaptopropionic acid (MPA) self-assembled monolayer (SAM) modified gold electrode. Parabenzoquinone (PBQ) was used as an electron mediator which is reduced to hydroquinone (H_2_Q). The EIS measurements showed that the charge transfer resistance (R_ct_) decreases with the increase of the glucose concentration due to the increase of the diffusion current density by the H_2_Q oxidation. The nondestructive and straightforward method showed a dynamic range of glucose determination with linear variation of the sensor response (1/R_ct_) as a function of glucose concentration in a solution [79]. Recently, versatile biosensing materials such as semiconducting CNTs and conducting polymers have been introduced [80]. Besteman *et al.* combined the GOx to the sidewall of the single-wall carbon nanotube (SWNT) by the use of a cross linker and found conductance decrease of the SWNT as well as the change of capacitance. The conductance increased upon exposure to glucose indicating that an enzyme-based single molecular level biosensor is available by the use of the SWNT [78]. Very few reports are available on nanostructured metal-oxide modified glucose sensor based on the conductometry or the EIS [81]. Ansari *et al.* covalently immobilized GOx on the SnO_2_ nanostructured thin film grown on anodized aluminum oxide (AAO) pores by the plasma enhanced chemical vapor deposition (PECVD) leading to the conductivity increase of the film. This film showed higher sensitivity towards glucose as compare to other simple nanostrucutres [82]. More improved sensitivity and wider linear response range could be available by tailoring the material properties, for example active surface area, three-dimensional structure, and electrical conductance of the film.

## 3. Glucose Sensor Based on Metal-Oxides

Metal-oxide based sensors are very sensitive, relatively inexpensive and have the advantage of rapid response associated with specific nanostructures such as nanowire, nanorod, nanotube, nanoparticle, nanofibre, CNT modified metal-oxide and so on. In this section, we will discuss recent development of enzymatic or nonenzymatic glucose sensors based on various nanostructured metal-oxides. It should be mentioned that during the last two decades tremendous efforts have been made for the detection of glucose based on nanostructured metal oxides and their composites. In Table 1 we tabulate metal oxides applicable to glucose sensors reported so far and give brief descriptions in terms of the detection methods, availability of enzymatic or nonenzymatic operations, sensitivity, detection limit, response time, and applied potential.

### 3.1. Zinc Oxide (ZnO) Based Glucose Sensor

ZnO nanomaterials have been studied extensively in optics, optoelectronics, sensors, and actuators owing to their semiconducting, piezoelectric, and pyroelectric properties [83]. ZnO has many attractive properties for the fabrication of the metal-oxide based biosensors, for example good biocompatibility, chemical stability, non-toxicity, electrochemical activity and fast electron transfer rate. Most of all, the ZnO substrate provides the GOx having many acidic protons with a better electrode surface for immobilization because the isoelectric point (IEP) of the ZnO is about 9.5, while that of the GOx is 4.2 [84,85]. Moreover, high surface-to-volume ratio of nanostructured ZnO gives the immobilized GOx a better electrical contact to the electrode [86,87]. Among many nanostrucuted ZnO, the ZnO nanorods have been widely studied for the immobilization of biomolecules [69,88,89]. Wei *et al.* introduced the ZnO nanorod grown on a gold electrode by hydrothermal decomposition followed by the iimobilization of GOx in phosphate buffer solution at pH 7.4. Negatively charged GOx in a neutral or basic solution is electrostatically immobilized on to the positively charged ZnO nanorod in the same solution by applying an anodic potential. The modified electrode showed a high and reproducible sensitivity in short response time and apparent Michaelis-Menten constant towards glucose oxidation was 2.9 mM [90].

**Figure 4.**
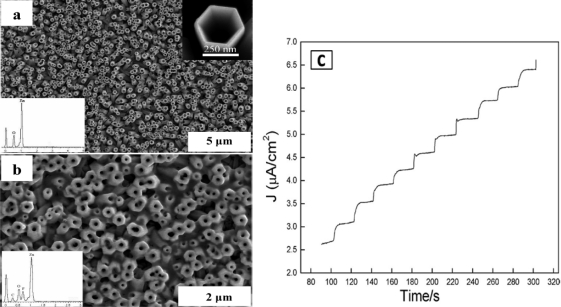
**(a)** Scanning electron microscope (SEM) image of the ZnO nanotube arrays; the energy dispersive X-ray spectroscopy (EDS) analysis (inset). **(b)** SEM image of the surface modified ZnO nanotube arrays; the EDS analysis (inset). **(c)** Typical amperometric response curve of GOx/ZnO nanotube arrays/ITO electrodes with the glucose concentration increases in 10 μM per step by successive addition of glucose to the 0.02 M phosphate buffer solution (PBS) at pH 7.4 under stirring. The applied potential was +0.8 V *vs*. SCE (reproduced with permission from [83]. Copyright 2009, The American Chemical Society).

Nanoporous ZnO and ZnO nanotubes have improved surface-to-volume ratios and also show highly sensitivity towards glucose oxidation. The porous ZnO:Co nanocluster having an average particle size of 5 nm showed much higher sensitivity (about 13.3 μA mM^−1^ cm^−2^) due to the high specific active sites and electrocatalytic activity of the ZnO:Co nanoclusters as well as strong affinity to the GOx [91]. Yang *et al.* synthesized porous ZnO nanotube arrays on ITO by two-step electrochemical and chemical processes (Figure 4). The Nafion/GOx/ZnO nanotube arrays on ITO electrode showed good mechanical contact between the ITO substrate and the ZnO nanotubes which leads to the improved sensitivity [83]. Similarly, improved sensitivity with the porous tetragonal pyramid-shaped ZnO nanomaterials and the ZnO nanocomb structure were also reported by other groups [92–94].

Physically or chemically tailored ZnO nanowires also lead to the high specific surface area and high IEP for efficient immobilization of concentrated GOx and the nanowire structure effectively mediates the electron transfer of the redox reaction [69]. Liu *et al.* developed a carbon-coated ZnO (C-ZnO) nanowire arrayed electrode by taking advantage of electrical conductivity and chemical stability of the carbon material and the one dimensional channel structure of ZnO nanowires [92]. The Nafion/GOx/C-ZnO nanowired electrode exhibited a pair of well-defined redox peaks at −0.43 and −0.48 V, resulted from the direct electron transfer between the immobilized GOx and the electrode. By contrast, no peaks were observed from both Nafion/GOx and Nafion/C-ZnO nanowired electrodes. Meanwhile, only very weak peaks are detected with a Nafion/GOx/pristine nanowired electrode. The EIS measurements confirmed the fast electron transfer at the C-ZnO nanowired electrode with charge transfer resistance of Fe[(CN)_6_]^3−/4−^ was ∼85 Ω, much smaller than that at the pristine nanowired electrode (∼400 Ω). The C-ZnO nanowire could be an inexpensive high-performing alternative to the conventional CNT-modified gold film in biosensor applications.

The nanostructured ZnO shows high sensitivity but very poor stability, because the ZnO nano-structure is easily removed from the electrode surface during functionalization [66,86]. Indeed, improved stability without loss of sensitivity or selectivity is one of the big challenges for glucose monitoring. Extensive efforts have been made to retain the catalytic activity of the immobilized GOx for a long time by using carbon-coated ZnO nanowire [95], functionalized CNTs [96], nanosized CaCO_3_ film [97], NdPO_4_ nanoparticle-chitosan composite [98], meldolas Blue mediated screen-printed electrodes [99] and many other materials. Wang *et al.* prepared ZnO nanoparticles and coated them onto a multiwall carbon nanotube (MWNT)-modified electrode for the GOx immobilization to improve the stability. A cationic polydiallyldimethylammonium chloride (PDDA) layer was further coated on the GOx-contained ZnO layer to prevent enzyme leakage [100]. The PDDA/GOx/ZnO/MWNTs film provided the sensing electrode with enhanced sensitivity, lower detection limit and long term stability more than 160 days. Results obtained from this glucose sensor were compared with one hundred human blood serum samples and agreed with the results of conventional spectrometric assay (correlation coefficient, 0.997).

In recent years, monitoring of intracellular glucose levels has received considerable attention and various methods including electrochemical detection [101], use of hypodermic needles [102], and microdialysis [103] have been proposed for the assessment of glucose in interstitial spaces as an alternative site rather than the bloodstream. Recently, Asif *et al.* developed a functionalized ZnO-nanorod based electrochemical sensor for the selective detection of glucose in human adipocytes and frog oocytes [104]. Hexagonal ZnO nanorods grown on the tip of a silver-covered borosilicate glass capillary make it possible to microinject specific reagents, which can interrupt or activate signal transmission to glucose, into relatively large adipocytes and oocytes. The prepared nanosensor exhibited a glucose-dependent electrochemical potential difference over the concentration range of 0.5–1000 μM *versus* a micro-sized Ag/AgCl reference electrode with a fast response time (<1 s). Intracellular and extracellular measurements of glucose showed good sensitivity and selectivity without interferences even though the nanosensor encountered stability problems.

Even if ZnO nanomaterials are highly promising electrode materials for glucose sensing, a relatively high potential is still required for operation meaning that unwanted output signal caused by the oxidation of interfering agents such as ascorbic acid (AA) or uric acid (UA), might be usually coexisted with glucose signal in real samples.

### 3.2. Copper Oxide (CuO/Cu_2_O) Based Glucose Sensor

Transition metal oxides and alloys significantly enhance direct oxidation of glucose compared with other metals that attribute to the catalytic effect resulting from the multi-electron oxidation mediated by surface metal oxide layers [105,106]. Transition metals such as Cu and Ni can oxidize carbohydrate easily without surface poisoning. Unlike Cu and Ni, corresponding oxides or hydroxides are relatively stable in air and solutions [107,108]. Natural abundance of copper oxide as well as its low production cost, good electrochemical and catalytic properties make the copper oxide to be one of the best materials for electrical, optical and photovoltaic devices, heterogeneous catalysis, magnetic storage media, gas sensing, field-emission emitters, lithium ion electrode and so forth [109–111].

Recent advances in nanoscience and nanotechnology have revealed the catalytic effect of copper oxide in relation to nonenzymetic glucose oxidation, voltammetric sensing of carbohydrates and hydrogen peroxide detection with ultra-sensitive response and good stability [112]. Wang *et al.* prepared Pd (IV)-doped CuO nanofibers (PCNFs) and CuO nanofibers via electrospinning on glassy carbon electrodes (GCE) and investigated the amperometric direct responses to glucose [113]. The PCNFs modified electrode showed excellent selectivity, reproducibility and stability. The Pd in the PCNFs, with lower electron occupancy in 3d orbital compared to the Cu, act as a Lewis acid for adsorption of polar glucose molecule a Lewis bases, via nucleophilic attack by non-bonded electron pairs. The longer resident time for the reactant species within the electrode-electrolyte interface, the more enhanced electrocatalytic activity towards the glucose oxidation [105,114]. A similar approach was also demonstrated by using three-dimensional network of CuO nanofibers (CuO-NFs) and reported improved sensitivity, stability and fast response time compared to the PCNFs modified electrode [115]. The CuO-NFs modified electrode also shows good selectivity and resistance to electrode fouling. Zhuang *et al.* developed a highly stable and sensitive nonenzymatic glucose sensor based on copper oxide nanowire modified Cu electrode in an alkaline medium [116]. The CuO nanowire can greatly increase the electrocatalytic active area and promote electron transfer rate of glucose oxidation. The CuO modified electrodes could be used repeatedly and were not contaminated with by-product of glucose oxidation. Experimental data for the glucose detection are in good agreement with the results from the spectrophotometric method performed in local hospital in real sample, where interference effect is negligible.

Recently, the existence of CuO nanoparticles as impurities in a CNT-based electrode has been claimed to be responsible for electrocatalytic activity of glucose oxidation [117] as the synthetic procedures of pure CuO nanowire or nanofiber are tedious and time consuming. In addition, air-sensitive copper substrate can make a big sensor-to-sensor variation when exposed to open air under most environmental conditions. Combination of CNTs with copper oxide nanoparticles enhances the electron transfer rate of Cu_2_O and are successfully applied as an enzyme-free glucose Sensor. Very recently, Zhang *et al.* and Jiang *et al.* have developed Cu_2_O/MWNTs and CuO/MWNTs modified nonenzymatic electrodes for glucose sensing [118,119]. The Cu_2_O/MWNTs nanocomposites were prepared on GCEs by a new fixure-reduction method at low temperature. It showed higher sensitivity and lower detection limit as compared to the CuO/MWNTs that attributes to the stability factor of those two different copper oxide films [120]. Very few reports are available on CuO based enzymatic glucose sensors for example, immobilization of CuO-GOx mixture within a carbon paste matrix [121] and immobilization of GOx on flower-shaped CuO nanostructured electrode [122].

### 3.3. Manganese Dioxide (MnO_2_) Based Glucose Sensor

Most enzymatic glucose sensors take advantage of the reducing power of hydrogen peroxide produced by the degradation of glucose via the GOx involved catalytic reaction. The oxidation of hydrogen peroxide is accompanied by the application of a bias potential at which, however, coexisting species such as AA are also electroactive [123]. Basically, two options are available to avoid the electrochemical activity of interfering agents. One is to employ a permselective membrane and the other one is to lower the applied potential by using electron mediators [124–129]. The permselective membrane may decrease the sensitivity and may not completely exclude the interfering effect. As an example of the permselective membrane, MnO_2_ a strong oxidant has been tested to get rid of interference signals in glucose sensing by Choi *et al.* [130]. The IEP of MnO_2_ is quite low (4–5) at pH ranging from 2.8 to 4.5 but at higher pH, MnO_2_ showed favorable environment for the immobilization of biomolecules [131,132].

**Figure 5.**
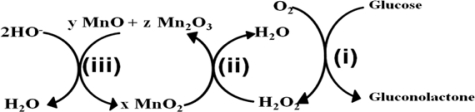
Reaction mechanism of glucose at a MnO_2_/GOx modified SPE based on heterogeneous carbon material: **(i)** enzymatic oxidation of glucose by GOx, **(ii)** chemical oxidation of H_2_O_2_, and **(iii)** chemical reduction of H_2_O_2_ (redrawn from reference [133]).

Turkusic *et al.* developed an amperometric glucose sensor based on carbon paste electrodes modified with MnO_2_ and GOx that operated at pH 9.5 [133]. They also demonstrated glucose degradation mechanism on the MnO_2_/GOx modified screen printed electrode (SPE) based on heterogeneous carbon material, as shown in Figure 5. The two enzymatic oxidation products, gluconolactone and H_2_O_2_ were produced by applying 0.48 V *vs.* Ag/AgCl. The H_2_O_2_ further reacts chemically with MnO_2_ to produce manganese species having lower oxidation states, which can be electrochemically reoxidized to form MnO_2_ and the oxidative current flow is directly proportional to the glucose concentration. This rapid electrochemical process is accompanied by a kinetically slower chemical reoxidation of MnO/Mn_2_O_3_ coupled with chemical oxidation of H_2_O_2_. The MnO_2_/GOx modified SPE showed partially decreased interfering signals, along with good reproducibility and long term stability.

Poly(diallyldimethylammonium), PDDA/MnO_2_ and chitosan/MnO_2_ nanocomposites are excellent electrode materials to minimize interference effect of UA and AA at low potential. Xu *et al*. fabricated a PDDA/MnO_2_ nanocomposite on graphite electrode surface at pH 7.0 a favorable pH for the GOx immobilization. As-prepared PDDA/MnO_2_/GOx modified electrode is free from interferants when operated at 0.46 V [134]. They also suggested chitosan film containing MnO_2_ nanoparticles where the GOx was entrapped to be immobilized into chitosan hydrogel and showed effective suppression of interfering signals of ascorbates [135].

Chen *et al.* developed a nonenzymatic electrochemical glucose sensor modified with MnO_2_/MWNTs [136]. The MnO_2_/MWNTs electrode displayed high electrocatalytic activity towards the glucose oxidation in alkaline solutions at 0.3V and also strongly resisted toward poisoning by chloride ions. In addition, the problematic interference that occurs by the oxidation of common interfering species such as AA, dopamine (DA), and UA was effectively suppressed in real sample analysis compare to the unmodified MWNTs.

### 3.4. Titanium Dioxide (TiO_2_) Based Glucose Sensor

TiO_2_ nanomaterials are basically biocompatible and environmentally-friendly and have been frequently proposed as a prospective interface for the immobilization of biomolecules [137,138], and widely applied in photochemistry [139–141] and electrochemistry [142,143]. Moreover, titanium forms coordination bonds with the amine and carboxyl groups of enzymes and maintains the enzyme’s biocatalytic activity. Nanostructured TiO_2_ also provides the enzyme with better immobilization environment by enlarging the surface area [144,145].

Sol-gel technology has been widely explored in the field of chemical sensors and biosensors. Especially, the low-temperature sol-gel process enables encapsulation of heat sensitive biomolecules such as enzymes, protein molecules and antibodies [146–149]. Various sol-gel derived TiO_2_ films associated with binding molecules or polymer networks for crack-free porous structures have been characterized and applied to biosensing [150–155]. Viticoli *et al.* developed a third generation amperometric glucose sensor based on nanostructured TiO_2_ on a porous silicon (p-Si) substrate. The Functionalized TiO_2_ layers were prepared by the metal organic chemical vapor deposition or by the sol-gel technique at various pressures (0.1–4 torr) and temperatures (300 °C to 800 °C) using Ti(IV) isopropoxide as a starting precursor [156]. Enzyme was directly dip-coated on the TiO_2_ modified p-Si substrate. Results showed a good linear response with a few seconds of response time.

Recently, Bao *et al.* have hydrothermally synthesized a new slack TiO_2_ layer having a uniform porous nanostructure by the use of MWNTs template [157]. The TiO_2_ nanostructure displayed a big hysteresis loop at high pressure. Abrupt increase of adsorption at high pressure in the nitrogen adsorption and desorption isotherms confirms the presence of porous structure. The porous TiO_2_ nanostructure has a large capacity for enzyme immobilization that was proved by observing a big change in shape of the hysteresis loop and a significant decrease of the specific surface area of the nanostructure. The applied potential was 0.45 V at pH 6.6 under a N_2_ atmosphere. Although the porous TiO_2_ nanostructure-modified GCE strongly depends on pH, it operates at low enough potential to eliminate interference signals of UA and AA.

Fluorescence based glucose sensors have appeared in the literature as an alternative way of continuous monitoring of glucose levels. These sensors are highly specific towards analytes but require built-in probes [158]. A number of reports on reagentless optical biosensors using pH-sensitive fluorescent probes are available [159–163]. Fluorometric detection of dissolved oxygen is available since the oxygen is able to quench the fluorescent probe. Doong *et al.* developed a novel optical arrayed TiO_2_ biosensor for simultaneous detection of anlaytes containing glucose, urea and glutamate in a serum sample [164]. Carboxyseminaphthorhodamine-1-dextran (SNARF-1-dextran), a single-molecule fluorescent probe, was co-immobilized with glucose dehydrogenase on TiO_2_ by the sol-gel process. The glucose dehydrogenase decomposes glucose to produce proton and the pH decreases. An enhanced sensitivity at basic condition is primarily attributed to the fluorescent characteristics of the carboxy SNARF-1-dextran that gives strong emission intensity at 630 nm. As-prepared TiO_2_ arrayed optical sensor showed still good sensitivity even after stored at 4 °C for 1 month.

### 3.5 Cerium Oxide (CeO_2_) Based Glucose Sensor

Nanostructured CeO_2_ is an excellent electrode material because it is a nontoxic, chemically inert and electrically conductive material. It also shows large surface area like other nanostructured materials and good biocompatibility [165–173]. Additionally, CeO_2_ can act as electrochemical redox couple that makes it possible to produce a mediatorless glucose sensor. High IEP (∼9.0) and electron-transfer rate constant (18.3 s^−1^) with a good surface coverage (1.4 × 10^−11^ mol cm^−2^) of CeO_2_ allow easier enzyme immobilization and direct electron transfer between the active sites of the GOx and the electrode surface [174,175].

**Figure 6.**
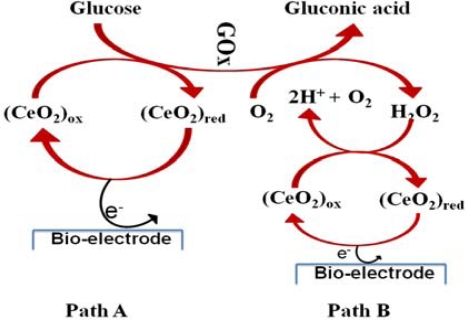
Schematic illustration of two possible biochemical reaction mechanisms on the GOx/CeO_2_/Pt electrode (redrawn from reference [176]).

Saha *et al.* deposited nanoporous CeO_2_ thin film on a Pt coated glass plate using pulsed laser deposition (PLD) [176]. GOx was immobilized on to the CeO_2_ by the electrostatic interaction between the positively charged CeO_2_ and the negatively charged GOx at pH 7. Prior to the enzyme immobilization the GOx/Pt electrode was pretreated electrochemically at 0.8 V to activate the CeO_2_ surface and to remove the oxidizable impurities. The resulting GOx/CeO_2_/Pt electrode showed a linear response to glucose oxidation with low Michaelis-Menten constant (1.01 mM) indicating enhanced enzyme affinity to glucose. The mechanisms available for the glucose oxidation on the electrode are depicted in Figure 6.

Path A describes direct electron transfer between the GOx and the electrode via oxidation and reduction of CeO_2_ in which the CeO_2_ is a better electron acceptor than oxygen. This is clearly confirmed by observing no oxidation peak of H_2_O_2_ [177], whereas, in path B, the electron generated by the glucose oxidation might be coupled with reduction of molecular oxygen followed by reduction of CeO_2_ and then finally transferred to the electrode at acidic condition [178]. Sol-gel derived nanostructured CeO_2_ film on Au electrode is also available for the GOx immobilization and suggested by Ansari *et al.* [179]. The GOx physically adsorbed on the CeO_2_/Au electrode showed a linear response in the range of 0.5 g L^−1^ to 4 g L^−1^. The detection limit was 12.0 μM with a shelf life of 12 days. Recently, various nanostructured CeO_2_ such as nanorod, nanocomb, nanocubes, and nanoflower [180,181] have been synthesized. However, no successful reports about glucose detection are available and the potential application of these nanostructures for glucose sensing is of great interest.

### 3.6. Silicon Dioxide (SiO_2_) Based Glucose Sensor

Basically, electrode materials should have good electron transport capacity, bioactivities towards target analytes and provide suitable physical or chemical environments for reliable immobilization of biorecognition molecules. Some metal oxides meet those requirements perfectly, but there are still demands for other nanostructured materials including conducting polymers and CNTs to get more enhanced sensitivity and reliability of a glucose sensor. Indeed, composite materials made from two or more nanostructures are another big issue [182–184].

Silica based organic and inorganic nanocomposites are attractive electrode materials since they provide biorecognition molecules with a better entrapment environment and more enhanced electrochemical stability [185]. A mesocellular silica–carbon nanocomposite foam (MSCF) was designed for the GOx immobilization by Wu *et al.* [186]. The uniformly ordered MSCF showed good biocompatibility, favorable conductivity and hydrophilicity. The narrow pore-size distribution was suitable for the immobilization of not only the GOx but also other redox proteins. A fast electron transfer rate (14.0 ± 1.7 s^−1^) of the GOx modified MSFC allowed direct electrochemistry and showed a good electrochemical sensitivity to glucose with a linear response range of 50 μM to 5.0 mM with a detection limit of 34 μM at an applied potential of −0.4 V. The low potential for operation prohibits unwanted interference signal in real sample by the oxidation of AA and UA.

Recently, extensive researches have been done on the immobilization of GOx by the use of silica nanocomposites such as unprotected Pt nanoclusters mixed with the nanoscale SiO_2_ particles [187], chitosan/SiO_2_ nanocomposites attached on the Pt-MWNTs modified electrode [188], TiO_2_/SiO_2_ nanocomposite [189], sol–gel silica film on a prussian blue modified electrode [190]. Porous SiO_2_ nanofiber is also available for the potentiometric biosensor application [191].

Gopalan *et al.* made a form of silica network on Nafion and subsequently loaded polyaniline grafted MWNTs (MWNTs-g-PANI) on the Nafion–silica nanocomposite for the GOx immobilization [192]. Nafion, silica, MWNTs, and conducting polymers have individually been known as materials for improving the electrocatalytic activity, electron conduction path, sensitivity, and stability of biosensors, respectively [193–195]. The Nafion–silica/MWNTs-g-PANI electrode exhibited a linear response to glucose in the range of 1 mM to 10 mM with a correlation coefficient of 0.9972. The sensitivity was 5.01 μA mM^−1^ with a low response time (∼6 s). Although the entire fabrication procedure is somewhat complicated and requires high fabrication cost, the Nafion–silica/MWNT-g-PANI composite electrode showed excellent sensing performance with negligible interference from AA, UA, and acetaminophen (AP). The recovery test of the Nafion–silica/MWNTs-g-PANI electrode in real sample was evaluated by the standard addition method, with three times addition of standard glucose solution. Experimental showed that reproducible current response (R.S.D) for three measurements were in the range of 2.9−4.1% with the recovery range of 98.0–105.5.

### 3.7. Zirconium Oxide (ZrO_2_) Based Glucose Sensor

Nanostructured ZrO_2_ is another example for the direct electron transfer between metal-oxide layer and the immobilized GOx for glucose sensing. Because the IEP of ZrO_2_ is of 4.15 [196], it is suitable for the adsorption of high IEP proteins. Therefore, any other nanomaterial, which can drop down the IEP of ZrO_2_ when it is mixed with ZrO_2_ is needed for the application of ZrO_2_-based electrode to glucose sensing since the IEP of glucose is of 4.2 [197]. The first amperometric glucose sensor based on sol-gel derived ZrO_2_ was reported by Liu *et al.* [198]. Since then, a numerous efforts have been reported for the development of nano ZrO_2_ based biosensors [199–201]. Yang *et al*. prepared ZrO_2_/Pt-PLL (poly-Lysine) and ZrO_2_/Pt-PVA (polyvinyl alcohol) to modify pyrolytic graphite (PG) electrodes for the GOx immobilization [202]. Didodecyldimethylammonium bromide (DDAB), a synthetic lipid commonly used as a bio-membrane was introduced to stabilize and protect inorganic nanoparticles. The resulting GOx/ZrO_2_/Pt-PVA electrode showed largest reaction activity towards glucose oxidation in the presence of ferrocenium hexafluorophosphate (FcPF_6_) as an electron transfer mediator. On the other hand, no enzymatic activity of the immobilized GOx can be observed on ZrO_2_/DMSO and ZrO_2_/DDAB film. So, the use of colloidal platinum by replacing DMSO and DDAB plays an important role in transferring electrons between GOx and the electrode.

Table 2 summarizes characteristics of the most frequently-used metal oxides what we have discussed in the section 3.1 to 3.7 in terms of EIP, the availability of enzymatic or nonenzymatic sensors, the compatibility with CNTs, conducting polymers or metal nanoparticles, and the application for other biosensors.

### 3.8. Other Metal-Oxide Based Glucose Sensors

Plenty other metal oxides such as NiO, SnO_2_, MgO, IrO_2_, WO_3_, PbO_2_, RhO_2_, IrO_x_, V_2_O_5_, Fe_3_O_4_, and RuO_x_ have been reported and their use, not only for glucose sensing, but also for many other biosensing applications to improve sensing performances attempted [203–213]. In this section we will briefly introduce some of those metal-oxide based sensors, including their applications and future prospects.

In recent years, NiO nanomaterials have received considerable attention due to their broad application to battery cathodes, gas sensors, electrochromic films, magnetic materials and catalysts [214,215]. However, there are only few biosensor applications. Li *et al.* developed a novel amperometric glucose sensor based on NiO hollow nanosphere [203]. The NiO is suitable for electrostatic immobilization of proteins having low IEP because the IEP of NiO is about 10.7. The hollow-sphered NiO was good responsible for high loading of GOx and showed fast electron transfer with a sensitivity of 3.43 μA mM^−1^ and a detection limit of 47 μM (S/N = 3).

MgO as a ceramic material has been typically applied to water purification, additives in refractory, paint, and superconductors. Various nanostructured magnesium oxides have been reported [216–218] but only a few reports are available for biosensor applications [219,205]. Umar *et al.* first introduced the application of MgO as an electrode material for glucose sensing [205]. Polyhedral nanocages and nanocrystals of MgO were grown on silicon substrates via non-catalytic simple thermal evaporation process or on a gold surface and used as immobilization matrices for GOx. The resulting Au/MgO/GOx/Nafion electrode showed a good stability and sensitivity of 31.6 μA μM^−1^ cm^−2^. The response time was less than 5 seconds.

Kotzian *et al.* reported an amperometric glucose sensor based on rhodium dioxide (RhO_2_) modified screen printed carbon electrode for the first time [209]. The GOx were mixed with equal amounts of nafion and then transferred to the RhO_2_ modified SPE. The flow injection analysis of glucose at an operating potential of 0.2 V *vs.* Ag/AgCl showed an excellent detection limit of 0.2 mg L^−1^ when the optimized flow rate was 0.4 mL min^−1^ in a 0.1 M phosphate buffer (pH 7.5) solution and the interference effect was surprisingly reduced because the operational potential was relatively very low.

## 4. Conclusion: Future Prospective and Challenges

Numerous efforts have been made to devise an ultrasensitive biosensor for monitoring blood glucose without interference from other electroactive species. With the advent of nanotechnology, the regulation of sensing devices at molecular level is possible to some extent. In spite of the impressive success of glucose monitoring by using various nanomaterials, however, there is still asking for continuous and non-invasive glucose sensing from diabetic patients in hospital with a much more reliable and sensitive glucose sensor. Since metal-oxide nanomaterials have basically large surface-to-volume ratio, high IEP values and provide a better surface for GOx immobilization, scientists have paid much attention to these electrode materials in anticipation of more stable, more sensitive but less interfering free glycemic monitoring. Additionally the metal-oxide based nonenzymatic glucose sensor enables cost-effective direct determination of blood glucose level without any electron transfer mediator and there is no loss of sensitivity due to denaturation of protein enzymes in the immobilization or detection procedures.

This review has briefly summarized the most frequently-used electrochemical methods and metal-oxides, including zinc oxide, copper oxides, manganese dioxide, titanium oxides, cerium oxide and silicon dioxide in glucose biosensors. ZnO and CeO_2_ having high IEP might be the most suitable electrode materials for glucose sensing. Various nanostructured ZnOs have been reported for glucose monitoring and they showed excellent sensitivity and selectivity. Mediatorless glucose sensing is also available with the CeO_2_. On the other hand, the nanostructured CuO modified electrode is capable of direct electrooxidation of glucose without enzyme immobilization and reduces the costs of the sensor fabrication. Moreover, CuO nanostructure-modified electrode showed highest sensitivity and lower detection limit compare to other metal oxide based glucose sensors and also showed less interference due to the low operating potential. Many other metal-oxides for example nickel and magnesium oxides are also available for glucose sensing. Some metal-oxides are highly sensitive but show poor stability and serious interference effect because of high potential requirement for operation. On the other hand, other metal-oxides operate at low enough potential not to produce interfering signals and stability problem either but their sensitivity is totally unsatisfactory. Nevertheless, glucose sensing based on novel metal-oxide nanomaterials still has many advantages for the glucose detection in terms of miniaturization and development of semi-invasive or finally non-invasive sensing devices especially for the *in vivo* detection even though it requires more academic and technical studies for commercialization. Indeed, growing research interest of glucose sensors will continue with increasing prevalence of diabetic patients and the theoretical background and the experimental expertise acquired through investigation of metal-oxide nanostructured glucose sensors will be extended to overall biosensor industry.

## Figures and Tables

**Table 1.** Metal-oxides and metal-oxide composites available for glucose sensors and their functional properties.

**Electrode matrix**	**Detection techniques**	**Enzymatic/nonenzymatic**	**Sensitivity/detection limit (μM)**	**Response time(s)/applied potential(V)**	**Ref.**

CuO nanospheres	Amperometric	nonenzymatic	404.53 μA mM^−1^ cm^−2^/1	−/+0.60	[12]
MOSFET using a ZnO nanowires	Potentiometric	enzymatic	−/∼10^−3^	−	[54]
n-type silicon substrates covered with SiO_2_ and/or Al_2_O_3_	Potentiometric	enzymatic	13 mV mM^−1^/−	−	[56]
ENFET doped with SiO_2_ nanoparticles	Potentiometric	enzymatic	48 mV pH^−1^ in the pH range of 2–12/25	300/−	[57]
ZnO nanowire	Amperometric	enzymatic	26.3 mA mA^−1^ cm^−2^/0.7	10/+0.8	[68]
ZnO nanorods	Amperometric	enzymatic	−/3	<5/−0.1	[69]
Nano-basket SnO_2_ templated in porous Al_2_O_3_	Conductmetric	enzymatic	−/in the range of 5 × 10^3^–2 × 10^4^	−	[82]
ZnO nanotube	Amperometric	enzymaitc	30.85 μA mM^−1^ cm^−2^/10	<6/+0.8	[83]
ZnO nanorod	Amperometric	enzymatic	23.1 μA mM^−1^ cm^−2^/10	<5/+0.8	[88]
ZnO:Co nanocluster	Amperometric	enzymatic	13.3 μA mM^−1^ cm^−2^/20	8/0.55	[91]
pyramid-shaped porous ZnO	Amperometric	enzymatic	−/10	−/−0.50	[92]
ZnO nanotube	Amperometric	enzymatic	21.7 μA mM^−1^ cm^−2^/1	3/+0.8	[93]
ZnO nanocomb	Amperometric	enzymatic	15.33 μA mM^−1^ cm^−2^/20	<10/+0.8	[94]
C-decorated ZnO nanowire	Amperometric	enzymatic	237.8 μA mM^−1^ cm^−2^/0.2	∼5/−0.45	[95]
MWNTs/ZnO nanoparticle	Amperometric	enzymatic	50.2mA cm^−2^ M^−1^/0.25	6/−0.1	[100]
Pd (IV)-doped CuO oxide nanofiber	Amperometric	nonenzymatic	1061.4 μA mM^−1^ cm^−2^/1.9 × 10^−2^	1/+0.3	[113]
CuO nanofibre	Amperometric	nonenzymaic	431.3 μA mM^−1^ cm^−2^/−	∼1/+0.4	[115]
CuO nanowire	Amperometric	nonenzymatic	0.49 μA μmol^−1^ dm^−3^/0.049	−/+0.33	[116]
Cu_2_O/MWCNTs nanocomposites	Amperometric	nonenzymatic	6.53 μA μmol^−1^ L^−1^/0.05	−/−0.2	[118]
MWNTs/CuO nanoparticle	Amperometric	nonenzymatic	2596 μA mM^−1^ cm^−2^/0.2	∼1/+0.4	[119]
flower-shaped CuO	Amperometric	enzymatic	47.19 μA mM^−1^ cm^−2^/1.37	<5/+0.58	[119]
MnO_2_	Amperometric	enzymatic	−/0.472	−/+0.48	[133]
MnO_2_/MWNTs nanocomposite	Amperometric	nonenzymatic	33.19 μA mM^−1^/28 × 10^3^	−/+0.3	[136]
TiO_2_ nanofilm	Amperometric	enzymatic	−/∼1	few second/−0.45	[156]
Nanostructured TiO_2_/CNT	Amperometric	enzymatic	0.3 μA mmol^−1^/−	<10/−0.45	[155]
Array-based TiO_2_	Optical	enzymatic	−/3.1–7.8	−	[164]
Nanostructured CeO_2_	Amperometric	enzymatic	0.00287 μA mg^−1^ dL^−1^ cm^−2^/12.0	−	[179]
SiO_2_–Carbon Nanocomposite	Amperometric	enzymatic	−/34	−/−0.4	[186]
Nano-SiO_2_ and “unprotected” Pt nanoclusters	Amperometric	enzymatic	3.85 μA mM^−1^/1.5	−/+0.6	[187]
TiO_2_/SiO_2_ nanocomposite	Phosphorescence	enzymatic	−/1.2 × 10^−4^	−	[188]
CNT/perfluorosulfonate ionomer–SiO_2_ nanocomposite	Amperometric	enzymatic	5.01 μA mM^−1^/0.1	∼6/+0.2	[192]
ZrO_2_ nanoparticle	Amperometric	enzymatic	−	−/+0.4	[202]
NiO hollow nanospheres	Amperometric	enzymatic	3.43μA Mm^−1^/47	∼8/+0.35	[203]
MgO polyhedral nanocages and nanocrystals	Amperometric	enzymatic	31.6 μA μM^−1^ cm^−2^/6.83 × 10^−2^ ± 0.02	<5/+0.58	[205]
Nitrocellulose, NC/PbO_2_	Amperometric	enzymatic	0.183 μA mM^−1^ / −	−/+0.7	[208]
RhO_2_ modified carbon Ink	Amperometric	enzymatic	64 μA mM^−1^ cm^−2^/1.11	28/−0.2	[209]
RuO_x_ –prussian blue	Amperometric	nonenzymatic	6.2 μA mM^−1^ cm^−2^/40	−	[213]
RuO_2_ modified Screen printed electrode	Amperometric	enzymatic	−/0.611	−/+0.5	[220]
Fe_3_O_4_ nanoparticle/Chitosan	Amperometric	enzymatic	9.3 μA mM^−1^ cm^−2^/500	∼5/−	[221]
Ferrocene-modified Fe_3_O_4_@SiO_2_ magnetic nanoparticles	Amperometric	enzymatic	−/3.2	−/+0.35	[222]
Si–SiO_2_–Si	Potentiometric	enzymatic	12 mV decade^−1^ in human urine/−	90	[223]
SnO_2_ film	Potentiometric	enzymatic	50 ± 2 ΔmV ΔpC^−1^/−	∼300	[224]

**Table 2.** Characteristics of most frequently-used metal oxides in prospective biosensors.

**Metal-oxides**	**IEP**	**Availability of enzymatic/nonenzymatic sensor**	**Compatibility with CNT**	**Compatibility with conducting polymer, metal nanoparticle**	**Application for other biosensors**	**Reference**
ZnO	9.5	available/ N/A	available	N/A, Co	H_2_O_2_, gas, cholesterol	[84,88,91,100,225]
CuO	6.5	available/available	available	N/A, Pd(IV)	H_2_O_2_, carbohydrates, gas	[112,113,118,119]
MnO_2_	4–5	available/available	available	available, N/A	ascorbic acid, H_2_O_2_, Li^+^	[133,134,135,226]
TiO_2_	3.9–8.2	available/N/A	available	available, Pt	H_2_O_2,_ DNA hybridization, gas	[137,157,227]
CeO_2_	∼9	available/ N/A	N/A	N/A	DNA hybridization, H_2_O_2_	[167,170–172,176]
SiO_2_	1.7–3.5	available/N/A	available	N/A	H_2_O_2_, biomolecules, urea, penicillin	[56,186,187,191]
ZrO_2_	4.15	available/ N/A	N/A	N/A	H_2_O_2_, gas	[196,200,228]

* N/A= Not available
